# Association of a single nucleotide polymorphism in neuronal acetylcholine receptor subunit alpha 5 (CHRNA5) with smoking status and with ‘pleasurable buzz’ during early experimentation with smoking

**DOI:** 10.1111/j.1360-0443.2008.02279.x

**Published:** 2008-09

**Authors:** Richard Sherva, Kirk Wilhelmsen, Cynthia S Pomerleau, Scott A Chasse, John P Rice, Sandy M Snedecor, Laura J Bierut, Rosalind J Neuman, Ovide F Pomerleau

**Affiliations:** 1 Department of Psychiatry, Washington University School of MedicineSt Louis, MO, USA,; 2 Department of Genetics and Neurology, Carolina Center for Genome SciencesChapel Hill, NC, USA,; 3 Department of Psychiatry, University of MichiganAnn Arbor, MI, USA; 4 Department of Biochemistry and Biophysics, University of North CarolinaChapel Hill, NC, USA

**Keywords:** Case–control design, chromosome 15, early smoking experiences, nicotinic alpha-5 receptor subunit, single nucleotide polymorphisms (SNPs), smokers and never-smokers

## Abstract

**Aims:**

To extend the previously identified association between a single nucleotide polymorphism (SNP) in neuronal acetylcholine receptor subunit alpha-5 (CHRNA5) and nicotine dependence to current smoking and initial smoking-experience phenotypes.

**Design, setting, participants:**

Case–control association study with a community-based sample, comprising 363 Caucasians and 72 African Americans (203 cases, 232 controls).

**Measurements:**

Cases had smoked ≥ five cigarettes/day for ≥ 5 years and had smoked at their current rate for the past 6 months. Controls had smoked between one and 100 cigarettes in their life-time, but never regularly. Participants also rated, retrospectively, pleasurable and displeasurable sensations experienced when they first smoked. We tested for associations between smoking phenotypes and the top 25 SNPs tested for association with nicotine dependence in a previous study.

**Findings:**

A non-synonymous coding SNP in CHRNA5, rs16969968, was associated with case status [odds ratio (OR) = 1.5, *P* = 0.01] and, in Caucasians, with experiencing a pleasurable rush or buzz during the first cigarette (OR = 1.6, *P* = 0.01); these sensations were associated highly with current smoking (OR = 8.2, *P* = 0.0001).

**Conclusions:**

We replicated the observation that the minor allele of rs16969968 affects smoking behavior, and extended these findings to sensitivity to smoking effects upon experimentation. While the ability to test genetic associations was limited by sample size, the polymorphism in the CHRNA5 subunit was shown to be associated significantly with enhanced pleasurable responses to initial cigarettes in regular smokers in an a priori test. The findings suggest that phenotypes related to subjective experiences upon smoking experimentation may mediate the development of nicotine dependence.

## Introduction

The likelihood of initiating and persisting in smoking, and ultimately of becoming nicotine dependent, are all influenced by genetic factors, as demonstrated by numerous twin [1–5], linkage [6–9] and candidate gene [10–12] studies. Recently, a whole genome association study by Bierut *et al.*[13] identified several common variants that appear to be associated with nicotine dependence in Caucasians. In that study, which featured increased single nucleotide polymorphism (SNP) coverage in regions containing potential candidate genes, the most significant associations were for SNPs in genes with functions related to nicotine metabolism and gamma aminobutyric acid (GABAergic) and glutamatergic neurotransmission, showing *P*-values less than 10^−4^ for SNPs in, among others, neurexin 1 (NRXN1) and neurexin 3 (NRXN3) as well as the neuronal acetylcholine receptor subunit alpha-5 (CHRNA5) precursor on chromosome 15. A subsequent report by the same group [14] noted that the alpha 5 polymorphism, rs16969968, a non-synonymous coding SNP in exon 4 of CHRNA5, causes an aspartic acid-to-asparagine substitution that alters the function of the nicotine receptor. The findings for the alpha-5 receptor were strengthened by a recent study by Berretini *et al.*[15], in which a highly correlated CHRNA5/CHRNA3 haplotype (cluster of linked genes) was shown to be associated strongly (*P* < 10^−6^) with smoking behavior (cigarettes smoked per day).

In an attempt to replicate and extend these findings, we focused our attention upon phenotype associations for the top 25 SNPs tested with nicotine dependence by Bierut *et al.* Before analysis, we decided to test rs16969968 a priori, and then examine the other 24 SNPs. Unlike the Bierut *et al.* study, in which both cases and controls were smokers, the current study involved a control group of life-time never-smokers who had taken at least one puff from a cigarette (to ensure that they had actively rejected cigarette smoking). Also unlike that study, our sample included a small, exploratory sample of African American participants in addition to the larger sample of Caucasians.

Because recent studies have implicated various nicotine receptor subunits in moderating nicotine's impact in drug-naive animals [16–18] and in humans [19,20], we decided to explore possible associations between rs16969968 and phenotypes involving reactivity to cigarettes during early smoking experimentation. We were guided by observations that individuals reporting greater positive early smoking experiences were more likely to be regular nicotine-dependent smokers [21–25]. Based on twin studies suggesting that at least 50% of the variability in smoking initiation is attributable to genetic factors [26,27], we hypothesized that some of these effects might be mediated by variations in nicotine receptor function, and in particular by the non-synonymous coding SNP rs16969968.

## Methods

### Recruitment

Candidates responding to advertisements in local (Ann Arbor, MI, USA) newspapers or flyers posted on bulletin boards around the community were interviewed initially by telephone and asked about health and smoking habits. Candidates who met preliminary criteria were scheduled for an individual interview at the University of Michigan Nicotine Research Laboratory. Cases were recruited first through a parent project of the study—a laboratory investigation [28] involving administration of nicotine to current smokers stratified on nicotine dependence using the Fagerström Test for Nicotine Dependence (FTND) [29] (high dependence: FTND = 4 versus low dependence: FTND < 4) and on depression using the Center for Epidemiological Studies–Depression (CES-D) [30] and a computerized version of the Composite International Diagnostic Interview (CIDI) [31][high depression: CES-D = 16 and/or CIDI life-time diagnosis of major depressive disorder (MDD) versus low depression (CES-D < 16) and no life-time CIDI diagnosis of MDD]. A supplement was awarded later to enable the project to join the National Institute on Drug Addiction (NIDA) Genetics Consortium and to permit recruitment of never-smokers who did not participate in the laboratory study but were otherwise comparable to the smokers; additional current smokers were also recruited to supplement the core sample of laboratory participants. Efforts were made to keep the cells for nicotine dependence (smokers only), depression and sex approximately equal in size; thus, the sample was not distributed randomly on these variables. For all participants, inclusion and exclusion criteria specified that candidates be between 25 and 65 years old; not be currently pregnant or nursing; have no current or previous diagnosis of psychosis, bipolar disorder, mania or suicide attempts; have no current (<6 months) use of psychoactive medication; have no recent consumption of alcohol beyond specified limits (15 drinks/week for women; 21 drinks per week for men) and have no diagnosis of alcohol abuse or current illegal drug use.

### Case–control definitions and assessment of early smoking experiences

Smokers were required to have smoked at least five cigarettes/day of ≥ 0.5 mg nicotine for ≥ 5 years and had been smoking at their current rate for the past 6 months, indicating current and sustained smoking. Never smokers were defined as having smoked between one and 100 cigarettes in their life-time without developing a pattern of regular smoking.

Early smoking experiences were assessed via the Early Smoking Experiences Questionnaire (ESE) [21] asking participants to rate, retrospectively, pleasurable sensations and unpleasant sensations experienced when they smoked their first cigarette. Pleasurable and unpleasant sensations (overall), pleasurable rush or buzz, relaxation, nausea, cough and dizziness were all rated on a scale of 1 (none) to 4 (intense).

### Genotyping

This study reports results from genotyping 25 previously implicated SNPs and 207 ancestrally informative markers. All samples and Affymetrix Kit Control DNA (Affymetrix, Santa Clara, CA, USA) were thawed to room temperature. Each sample's concentration was measured using a Nanodrop ND-1000 spectrophotometer (Thermo Fisher Scientific, Pittsburgh, PA, USA), and an aliquot diluted immediately with ddH_2_O to 150 µg/ml (18.4 µl) into a single well of a barcoded 96-well plate. Samples were processed in this manner sequentially, until all 535 samples were diluted and distributed. Samples were arranged as 23 samples per plate. Twenty-four plates were used to accommodate 535 samples and 24 internal Affymetrix control DNA samples (one per plate), with an additional 17 wells available for 15 randomly selected duplicate samples included to measure concordance rates. All plates were prepared in one afternoon, sealed, and then frozen at −20°C until processed.

After all 24 plates were processed and scanned, the overall marker call-rates for each sample were ranked. [Call-rate is the proportion of genotypes for a given SNP that can be confidently interrogated, or ‘called’ (e.g. AA, AB, BB) based on a plot of the raw output from the genotyping assays in which genotypic classes are represented by a cluster of distinct data points.] The 23 poorest performing samples [determined by quality control (QC) call rate %] were removed, stock DNAs thawed and distributed onto a final plate, and then processed. A total of 598 experiments were performed on all samples, sample duplicates and controls over the entire genotyping effort. Scanned chip feature intensities were analyzed using the Affymetrix GeneChip Targeted Genotyping Analysis Software (TGAS) package, which normalized the data and made the individual marker genotype calls for all samples.

### Data cleaning and quality control

In order to verify self-reported ancestry and assess admixture within racial groups, we used the software Structure version 2.1 [32], which implements a model-based clustering method for inferring population structure using genotype data for an unlinked subset of the markers selected because the minor allele frequencies for each population are characteristic of ancestry. There were 11 participants who self-reported mixed ancestry, reported their primary ancestry inconsistently or did not report ancestry. Of the 11 participants in question, one was excluded for a low QC call rate, five were determined to be of mixed heritage (<80% ethnic identity) and were excluded, and five were found to exceed the 80% threshold and were included. Because the population definitions became more stringent with the exclusion of participants with measured admixture, two individuals previously measured >80% fell below this threshold and were excluded. Ultimately, 11 Asians, 13 Hispanics and four people with ambiguous ethnicity were not analyzed further because of small sample size for these groups. Three pairs of individuals were found to be related through the program Relpair version 2.0.1 [33] and were determined subsequently to be siblings; in each case, the one with the most complete data was retained.

Call rates averaged over 97% for all samples, and most samples were called at 98% or better. Seven of 598 experiments failed to reach the 90% call rate threshold but only four participant samples failed to reach the 90% threshold in at least one experiment. These four participants were removed from further analysis. The concordance rate for the 34 repeated samples (36 experiments; two samples repeated three times) was 99.3%. Call rates for individual markers were established by the TGAS program for call rates over all cases and controls. Call rates were excellent, with an average call rate of 99.64% for the genotyped markers including all ancestrally informative markers (AIMs). Only one AIM failed to achieve a QC call rate of 90% and was removed from further analysis. Hardy Weinberg equilibrium (HWE) tests were carried out to reduce the likelihood of spurious genotype associations resulting from technical artifacts from the genotyping platform. All markers in controls were deemed to be in equilibrium (*P* > 0.01); tests were carried out within race groups.

### Statistical analysis

SNP–phenotype associations were tested in logistic regression models implemented in SAS version 9.1. Genotypic effects were modeled as the additive effect of the number of minor alleles carried, and adjusted for race and gender. Analyses were also performed separately within the two racial groups. To control for multiple comparisons, we computed the false discovery rate (FDR) [34] for each of the 25 observed *P*-values. Briefly, this procedure uses the ranking of the observed *P*-values, the number of tests preformed and the hypothesized proportion of true null tests to determine the FDR or Q-value, defined as the expected proportion of tests called significant at a given *P*-value that would be false positives.

The four-level early smoking experience variables for each of the sensations were dichotomized into ‘none’ or ‘slight’ versus ‘moderate’ or ‘intense’. Phenotypic data were analyzed in SAS using χ^2^ and analysis of variance (ANOVA) as appropriate.

## Results

The analyzed sample included 363 Caucasians and 72 African Americans. The 25 most significantly associated SNPs from the Beirut *et al.* study were typed in this sample and analyzed in both racial groups. Table 1 shows participant characteristics and smoking history. For both cases (regular smokers) and for controls (never-smokers), Caucasians had greater years of education compared with African American participants. Among cases, number of cigarettes smoked per day and years of regular smoking were greater, and regular smoking began at a younger age in Caucasians compared with African Americans.

**Table 1:** Participant characteristics and smoking history.

	Cases (smokers; n = 203)		Controls (never-smokers; n = 232)		
			
	Caucasians (n = 155)	African Americans (n = 48)	Caucasians (n = 208)	African Americans (n = 24)	χ^2^ or F-test (P-value)
Age (years) (mean ± SEM)	38.4 ± 0.84	37.2 ± 1.51	40.2 ± 0.72	34.3 ± 2.14	Status: *F* = 0.16 (0.6881)
					Race: *F* = 6.15 (0.0135)
					S × R*: *F* = 2.77 (0.0967)
Sex (% female)	48%	35%	55%	63%	Status: χ^2^ = 1.69 (0.1932)
					Race: χ^2^ = 0.45 (0.5015)
					S × R*: χ^2^ = 0.22 (0.1364)
Education (years) (mean ± SEM)	14.3 ± 0.16	13.3 ± 0.28	16.3 ± 0.14	15.2 ± 0.40	Status: *F* = 54.6 (0.0001)S × R
					Race: *F* = 15.14 (0.0001)
					*: *F* = 0.16 (0.6875)
Age 1st try (mean ± SEM)	14.7 ± 0.40	17.1 ± 0.73	15.9 ± 0.36	15.6 ± 1.06	Status: *F* = 0.07 (0.7888)
					Race: *F* = 2.25 (0.1341)
					S × R*: *F* = 3.78 (0.0524)
No. cigs/day (mean ± SEM)	18.0 ± 0.69	13.7 ± 1.26			Race: *F* = 8.99 (0.0031)
Age of regular smoking (mean ± SEM)	17.4 ± 0.40	19.5 ± 0.70			Race: *F* = 6.53 (0.0114)
Years of regular smoking (mean ± SEM)	18.4 ± 0.96	12.8 ± 1.17			Race: *F* = 8.16 (0.0047)

SEM: standard error of the mean;

* S × R: status × race interaction effect.

Table 2 shows smoking-status associations for the 25 top SNPs from the study by Beirut *et al.*[13], comparing cases and controls in the present study. As noted earlier, we decided to first test rs16969968 to avoid the need for multiple testing corrections, and then test the other 24 SNPs. Although the other associations did not achieve significance after correction for multiple testing [β = 0.40 to detect an odds ratio (OR) of 1.5 for a SNP with a minor allele frequency (MAF) of 25% at α = 0.05 in the combined, race-adjusted sample], *P*-values for all the SNPs tested are listed in Table 2 as a guide for further research. The key finding is that, after adjustment for gender and race, rs16969968 was associated significantly with smoking status in Caucasians (OR = 1.51, *P* = 0.01) and in Caucasians plus African Americans (combined sample OR = 1.48, *P* = 0.01). Although Beirut *et al.* presented evidence suggesting that rs16969968 ‘A’ (smoking-risk) alleles act recessively, significant association was based on genetic effects modeled as the additive effect of minor alleles in the present study.

**Table 2:** Associations between current smoking and replication set single nucleotide polymorphisms (SNPs).

				Odds ratio (95% confidence interval)			P-value		
					
SNP	Chr	Position (BP)	Gene	Combined	Caucasian	African American	Combined	Caucasian	African American
rs7877	1	167986548	FM01	1.13 (0.84–1.53)	1.2 (0.87–1.65)	0.78 (0.32–1.9)	0.41	0.26	0.58
rs16864387	1	168015501	FM04	1.01 (0.7–1.43)	1.17 (0.75–1.81)	0.7 (0.38–1.3)	0.99	0.49	0.26
rs12623467	2	51136740	NRXN1	1.17 (0.94–1.46)	1.16 (0.87–1.55)	1.14 (0.81–1.6)	0.15	0.3	0.44
rs2767	2	233108318	CHRND	1.14 (0.85–1.52)	1.14 (0.83–1.57)	1.07 (0.49–2.32)	0.38	0.4	0.86
rs2276560	2	233276424	CHRNG	1.08 (0.8–1.45)	0.97 (0.7–1.33)	3.7 (0.97–14.06)	0.63	0.84	0.05
rs6772197	3	51126839	GRM2	0.85 (0.6–1.21)	0.88 (0.58–1.31)	0.65 (0.3–1.41)	0.36	0.52	0.28
rs3762611	4	46838216	GABRA4	0.96 (0.59–1.6)	0.91 (0.51–1.61)	1.2 (0.45–3.2)	0.88	0.75	0.71
rs2304297	5	135717335	TRPC7	0.87 (0.63–1.2)	0.79 (0.56–1.12)	1.86 (0.63–5.47)	0.38	0.18	0.26
rs510769	6	154454133	OPRM1	1.08 (0.79–1.47)	1.09 (0.78–1.53)	1.03 (0.47–2.26)	0.61	0.61	0.95
rs6320	7	154300269	HTR5A	0.97 (0.72–1.31)	0.91 (0.66–1.26)	1.33 (0.63–2.81)	0.86	0.57	0.45
rs2302673	8	42727356	CHRNA6	0.95 (0.68–1.34)	0.81 (0.55–1.21)	1.88 (0.83–4.3)	0.78	0.31	0.13
rs12380218	9	77165214	VPS13A	1.02 (0.73–1.43)	0.97 (0.69–1.36)	3.05 (0.72–13)	0.91	0.85	0.13
rs10508649	10	22902288	PIP5K2A	0.79 (0.36–1.73)	0.42 (0.07–2.6)	0.81 (0.33–1.96)	0.56	0.35	0.64
rs4142041	10	68310957	CTNNA3	1.16 (0.87–1.54)	1.12 (0.83–1.51)	1.82 (0.58–5.67)	0.32	0.44	0.3
rs4245150	11	112869857	DRD2	1.09 (0.82–1.44)	1.02 (0.77–1.37)	2.54 (0.76–8.48)	0.56	0.86	0.13
rs3021529	12	61831947	AVPR1A	1.02 (0.68–1.54)	0.96 (0.62–1.48)	1.71 (0.47–6.24)	0.91	0.84	0.42
rs17041074	12	107794340	DAO	1.01 (0.75–1.38)	1.07 (0.76–1.5)	0.75 (0.36–1.57)	0.92	0.69	0.45
rs16969968	15	76669980	CHRNA5	1.48 (1.08–2.03)	1.51 (1.1–2.07)	0.68 (0.11–4.37)	0.01	0.01	0.69
rs578776	15	76675455	CHRNA5	0.87 (0.64–1.2)	0.79 (0.56–1.12)	1.19 (0.61–2.32)	0.39	0.19	0.61
rs3813567	15	76721606	CHRNB4	0.87 (0.61–1.2)	0.84 (0.57–1.24)	1.07 (0.49–2.35)	0.41	0.38	0.86
rs4802100	19	46187865	CYP2D6	0.83 (0.49–1.43)	0.88 (0.51–1.5)	NA	0.5	0.63	NA
rs2673931	19	53599281	GRIN2D	0.76 (0.57–1.01)	0.74 (0.54–1)	0.84 (0.37–1.87)	0.06	0.05	0.66
rs6045733	20	1898858	PDYN	0.83 (0.63–1.1)	0.87 (0.65–1.17)	0.59 (0.23–1.53)	0.2	0.36	0.28
rs6517442	21	38211816	KCNJ6	1.08 (0.81–1.44)	1.13 (0.83–1.53)	0.82 (0.39–1.75)	0.59	0.43	0.61
rs1206549	22	17590414	CLTCL1	0.96 (0.68–1.37)	1.05 (0.7–1.56)	0.69 (0.32–1.49)	0.84	0.82	0.35

NA: not available.

The MAF of rs16969968 differed significantly between racial groups, with 174 Caucasians having one ‘A’ allele and 45 having two alleles, while only six African Americans carried one copy of the ‘A’ allele. Accordingly, this SNP had a negligible effect on risk of smoking in the African American sample in the race-stratified analysis. Moveover, in four of the 25 SNPs, major and minor alleles were reversed in Caucasians and African Americans.

In addition, minor alleles at rs16969968 were associated significantly with moderate-to-intense pleasurable rush or buzz in early smoking (OR = 1.6, *P* = 0.01) in Caucasians; these sensations in turn were associated highly with increased risk of current smoking (OR = 8.2, *P* < 0.0001). Further, moderate-to-intense pleasurable rush or buzz significantly predicted smoker/never-smoker status in a combined, race-adjusted analysis (OR = 6.2, *P* < 0.0001). No other initial smoking experience was associated significantly with the rs16969968 genotype.

Table 3 provides further characterization of early-experience patterns for respondents reporting moderate-to-intense reactions. Cases (regular smokers) exhibited enhanced sensitivity to cigarette smoking, with a larger proportion reporting more positive and more negative experiences than controls (never-smokers). With respect to race differences, only global displeasurable and relaxation effects reached significance in comparisons that took into account smoking status; in comparisons limited to just smokers, a significantly larger proportion of African Americans endorsed global pleasurable (*P* < 0.002) and global displeasurable experiences (*P* < 0.003), along with pleasurable rush or buzz (*P* < 0.002), dizziness (*P* < 0.004), relaxation (*P* < 0.001) and difficulty inhaling (*P* < 0.02).

**Table 3:** Proportion reporting moderate or intense responses (≤3) to early experimentation with smoking.

	Cases (n = 203)		Controls (n = 232)		
			
	Caucasians (n = 155)	African Americans (n = 48)	Caucasians (n = 208)	African Americans (n = 24)	χ^2^ (P-value)
Global pleasurable	30%	47%	7%	5%	Status: χ^2^ = 23.2 (0.0001)
					Race: χ^2^ = 0.11 (0.7390)
					S × R*: χ^2^ = 0.97 (0.3259)
Global displeasurable	38%	30%	64%	37%	Status: χ^2^ = 20.6 (0.0001)
					Race: χ^2^ = 5.11 (0.0238)
					S × R*: χ^2^ = 1.55 (0.2131)
Pleasurable rush or buzz	43%	62%	10%	6%	Status: χ^2^ = 39.7 (0.0001)
					Race: χ^2^ = 0.32 (0.5730)
					S × R*: χ^2^ = 1.48 (0.2232)
Dizziness	61%	45%	17%	16%	Status: χ^2^ = 56.9 (0.0001)
					Race: χ^2^ = 0.01 (0.9056)
					S × R*: χ^2^ = 0.53 (0.4649)
Nausea	28%	20%	18%	11%	Status: χ^2^ = 3.91 (0.0479)
					Race: χ^2^ = 0.69 (0.4054)
					S × R*: χ^2^ = 0.04 (0.8440)
Relaxation	27%	45%	4%	0%	Status: χ^2^ = 25.5 (0.0001)
					Race: χ^2^ = 25.5 (0.0001)
					S × R*: χ^2^ = 34.87 (0.0001)
Coughing	46%	33%	50%	53%	Status: χ^2^ = 0.54 (0.4628)
					Race: χ^2^ = 0.05 (0.8276)
					S × R*: χ^2^ = 1.06 (0.3038)
Difficulty inhaling	48%	31%	58%	47%	Status: χ^2^ = 3.04 (0.0814)
					Race: χ^2^ = 0.77 (0.3816)
					S × R*: χ^2^ = 0.22 (0.6392)

* S × R: status × race interaction effect.

## Discussion

The CHRNA5 subunit has been implicated as a potential risk factor for nicotine dependence [13] resulting from an amino acid variant in a highly conserved region of the intracellular domain [14]. The present results substantiated these observations and extended the findings for dependent and non-dependent smokers in the Bierut *et al.* study [13] to nicotine-dependent regular smokers versus never-smokers. A recent report by Berretinini *et al.*[15] of association between a CHRNA5/CHRNA3 gene cluster and number of cigarettes smoked per day—a different smoking phenotype—provides further support for the influence of the alpha-5 subunit on smoking across different contrasts. Specifically, in the Bierut *et al.* study, controls were current smokers with FTND scores of zero; in the Berretinini *et al.* study, controls were individuals (both current smokers and never-smokers) consuming fewer than five cigarettes per day; in the present study, controls were never-smokers who had tried at least one cigarette but had subsequently smoked fewer than 100 cigarettes in their life-time.

Differences in sensitivity to cigarettes during early experimentation may help to explain the disparate trajectories of individuals who go on to become regular smokers and those who do not. While our ability to reach conclusions about genetic associations with all variables of interest was limited by sample size, the gene-association analysis in the present study indicates that minor alleles at rs16969968 may have contributed to smoking by enhancing the reinforcing effects of nicotine in nicotine-naive individuals who went on to become regular smokers. Moreover, smoking-experience analyses indicate that these effects involved both positive and negative experiences. Taking into account other recent studies, the findings suggest that early smoking experiences may mediate nicotine dependence, involving not only alpha-5 subunits but possibly also beta-2 subunits [19] as well as alpha-6 and beta-3 subunits [20]. Entrainment of nicotine dependence via enhanced sensitivity to nicotine also provides a plausible mechanism for explaining findings from three closely related investigations showing that risk of smoking and lung cancer is associated with a group of genes in chromosome 15 coding for alpha-3, alpha-5 and beta-4 nicotine receptor subunits [35–37]. In the aggregate, the nicotine receptor findings provide critically needed genotype specificity for twin studies, indicating that at least 50% of the variability in smoking initiation is due to genetic factors [26,27].

The exploration of relationships between smoking status and early smoking experiences in the African American sample is inconclusive due to the small number of participants available. The following general observations can be made: in statistical contrasts of race and smoking status in regular smokers (cases) and controls (never-smokers), reports of relaxation during early experimentation with cigarettes were significantly greater in African Americans. Limiting the analysis to regular smokers provided even stronger support for the possibility that African Americans may be more reactive to initial cigarettes than Caucasians (manuscript in preparation). The fact that allele frequencies for the risk polymorphism (rs16969968) were substantially lower in the African American sample, however, makes it unlikely that this particular polymorphism can account for differences between African Americans and Caucasians in initial sensitivity to cigarettes, but the possibility that other nicotine receptor subunits (e.g. beta-2, beta-3 or alpha-6) are involved merits consideration. Systematic exploration of race differences in susceptibility to smoking in larger samples has the potential to contribute important new insights about nicotine dependence etiology.

The present study utilized retrospective reports of early experiences with smoking as supplemental phenotypic measures. Regular smokers clearly differed from never-smokers on these measures; moreover, several lines of evidence suggest that these differences are not attributable solely to selective recall of positive experiences in smokers and/or selective recall of negative experiences in never-smokers. (i) Greater sensitivity to initial cigarettes by people who subsequently become smokers has been demonstrated in a wide variety of individuals, from American adults [21] to Chinese adolescents [23]. (ii) As time from onset of smoking is more recent in adolescents than adults, demonstration of similarly enhanced sensitivity in young smokers suggests that the early-experience reports are not merely the result of recall distortion over time [22–24]. (iii) Systematic examination of test–retest reliability of subjective reactions to early smoking [38], including head rush or buzz, led to the conclusion that reliability was acceptable when response options are dichotomized (as in the present study). (iv) Using a period of abstinence to allow for dissipation of tolerance as a model for initial sensitivity, we found that retrospective reports of head rush or buzz during early experimentation with smoking predicted significantly which individuals rated re-exposure to nicotine administered via nasal spray as more pleasurable [25]. (v) Studies of various other aspects of smoking behavior [38,39] suggest that self-report of tobacco use is generally reliable.

The genetic findings of the present study, along with those of Ehringer *et al.*[19] and Zeiger *et al.*[20], implicate initial sensitivity as a key variable in smoking development [40] and provide critical support for the usefulness of early-experience reports. What is needed next is the development of real-time assessments of sensitivity to nicotine based on objective measures [41] that can be refined and validated ultimately as phenotypes in successive tests of association with genetic variants.

## Conclusion

This study provides new information about a genetic risk factor in the initiation and entrainment of smoking. The results of the gene-association tests suggest that a polymorphism in the CHRNA5 subunit modulates the expression of the initial smoking experience in nicotine-naive individuals. The findings suggest that phenotypes related to early subjective experiences may mediate nicotine dependence.

### Declarations of interest

Drs L. J. Bierut and J. P. Rice are listed as inventors on a patent (US20070258898) held by Perlegen Sciences, Inc., covering the use of certain SNPs in determining the diagnosis, prognosis, and treatment of addiction. Drs L. J. Bierut, C. S. Pomerleau and O. F. Pomerleau have also acted as consultants for Pfizer, Inc. in 2008.
